# Heterogeneous Flagellar Expression in Single Salmonella Cells Promotes Diversity in Antibiotic Tolerance

**DOI:** 10.1128/mBio.02374-21

**Published:** 2021-09-28

**Authors:** Zhihui Lyu, Angela Yang, Patricia Villanueva, Abhyudai Singh, Jiqiang Ling

**Affiliations:** a Department of Cell Biology and Molecular Genetics, The University of Maryland, College Park, Maryland, USA; b Department of Electrical and Computer Engineering and Biomedical Engineering, University of Delaware, Newark, Delaware, USA; Yale School of Medicine

**Keywords:** single cell, antibiotic tolerance, flagella, pathogenesis, *Salmonella*

## Abstract

Phenotypic heterogeneity among single cells in a genetically identical population leads to diverse environmental adaptation. The human and animal pathogen Salmonella enterica serovar Typhimurium exhibits heterogeneous expression of virulence genes, including flagellar and Salmonella pathogenicity island (SPI) genes. Little is known about how the differential expression of flagellar genes among single cells affects bacterial adaptation to stresses. Here, we have developed a triple-fluorescence reporter to simultaneously monitor the expression of flagellar and SPI-1 pathways. We show that the two pathways cross talk at the single-cell level. Intriguingly, cells expressing flagella (*fliC*-ON) exhibit decreased tolerance to antibiotics compared to *fliC-*OFF cells. Such variation depends on TolC-dependent efflux pumps. We further show that *fliC*-ON cells contain higher intracellular proton concentrations. This suggests that the assembly and rotation of flagella consume the proton motive force and decrease the efflux activity, resulting in antibiotic sensitivity. Such a trade-off between motility and efflux highlights a novel mechanism of antibiotic tolerance.

## INTRODUCTION

Genetically identical individuals living in the same microenvironment may exhibit different phenotypes. In recent years, such phenotypic heterogeneity has become a major research focus covering studies ranging from the sources of gene expression noise to the resulting functional consequences (1–4). One of the best-known examples of phenotypic heterogeneity is antibiotic tolerance, which allows a subpopulation of bacterial cells to survive transient antibiotic exposure (5, 6). Tolerance has become a significant cause of antibiotic failure in clinics and has been actively targeted to improve the treatment of bacterial infections (7). Furthermore, drug tolerance has been shown to promote the evolution of permanent antibiotic resistance (8, 9), which exacerbates the urgent threat of drug-resistant microbial infections. Antibiotic tolerance has been mostly associated with slow growth or low metabolism (10–12), although the underlying mechanisms remain elusive.

Salmonella enterica serovar Typhimurium is a bacterial pathogen in animals and humans and causes tens of millions of gastrointestinal infections in humans worldwide each year (13–15). In addition to gastroenteritis, *S*. Typhimurium and other nontyphoidal Salmonella strains also cause invasive human disease that often leads to death (14). Among the major virulence genes of *S*. Typhimurium are Salmonella pathogenicity island (SPI) and flagellar genes (16, 17). SPI-1 and SPI-2 genes are important for invasion of host cells and intracellular survival, respectively (18). Flagella are critical for Salmonella to move toward nutrients as well as within the host (19). In addition, the flagellin protein FliC stimulates the host immune response by activating caspase-1 in macrophages (15). The assembly and rotation of flagella both require proton motive force (PMF) and are energetically costly (19, 20). It has been shown that shutting off flagellar expression helps Salmonella cells evade the host immune response (21) and improve growth in culture (22). Interestingly, flagellar and SPI-1 expression is heterogeneous in Salmonella (23–27). The expressions of flagellar and SPI-1 pathways are interconnected at the population level (28). In this study, we constructed a triple-fluorescence reporter to detect SPI-1 and flagellar expression simultaneously and found that the expression of these two pathways was positively correlated among single *S.* Typhimurium cells. We next applied this reporter to study the functional impact resulting from the heterogeneous expression of flagellar and SPI-1 genes. To our surprise, we found that *fliC*-OFF cells displayed improved tolerance to bactericidal antibiotics compared with *fliC-*ON cells, in a manner independent of SPI-1 genes. We further show that the heterogeneous responses of *fliC-*ON and *fliC*-OFF cells to antibiotics depend on the efflux activity driven by PMF. Our results suggest that the expression and rotation of flagella compete with the efflux process for PMF. This trade-off leads to lower efflux activity and drug tolerance in cells expressing flagellar genes.

## RESULTS

### Cross talk between SPI-1 and flagellar pathways in single Salmonella cells.

**FIG 1:**
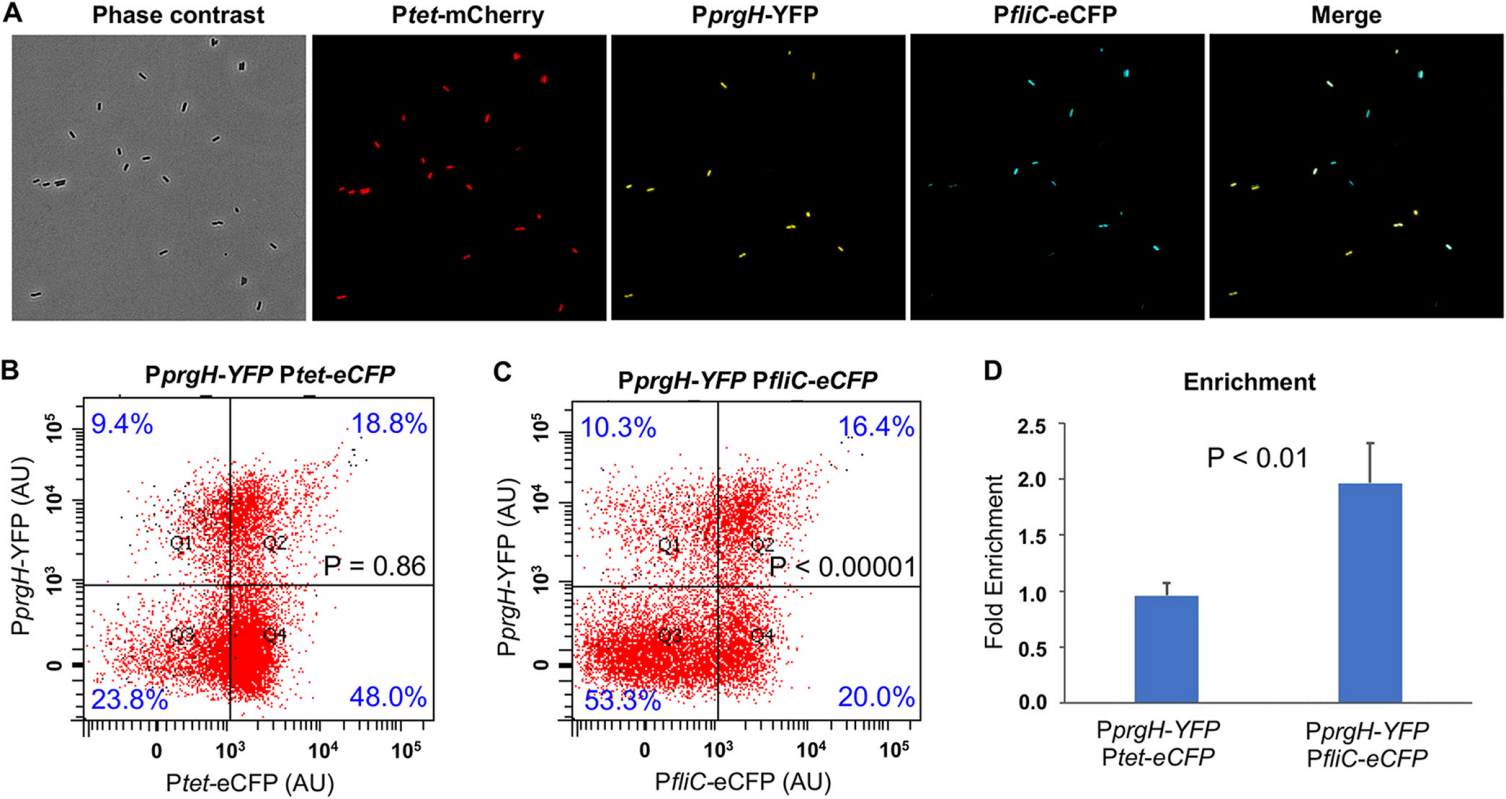
SPI-1 and flagellar expression are correlated in single Salmonella cells. (A) Phase-contrast and fluorescence microscopy of WT *S*. Typhimurium (ATCC 14028s) carrying pZS P*tet*-*mCherry* P*prgH-YFP* P*fliC-eCFP*. Cells were grown in LB Miller at 37°C for 5 h to the early stationary phase prior to imaging. While the constitutive P*tet-mCherry* promoter was expressed in all cells, only a fraction of cells were positive for P*prgH-YFP* or P*fliC-eCFP*. (B and C) Flow cytometry analysis of WT *S*. Typhimurium with pZS P*tet*-*mCherry* P*prgH-YFP* P*tet-eCFP* or pZS P*tet*-*mCherry* P*prgH-YFP* P*fliC-eCFP*. Cells were cultured under the same conditions as the ones described above for panel A. A significantly higher percentage of *fliC-*ON cells were *prgH*-ON than *fliC-*OFF cells. The data here are representative of results from at least four biological replicates. The *P* values were determined using the χ^2^ test (*n* = ∼10,000). AU, arbitrary units. (D) Fold enrichment was calculated as the percentage of *prgH*-ON cells among eCFP-ON cells divided by the percentage of all *prgH-*ON cells in the whole population. The probability of *prgH*-ON among the *fliC-*ON cells is 2-fold higher than for the overall population and 4-fold higher than for the *fliC-*OFF cells. Error bars represent 1 standard deviation (SD) from the mean (*n* = 4). The *P* value in panel D was determined using the unpaired *t* test.

**FIG 2:**
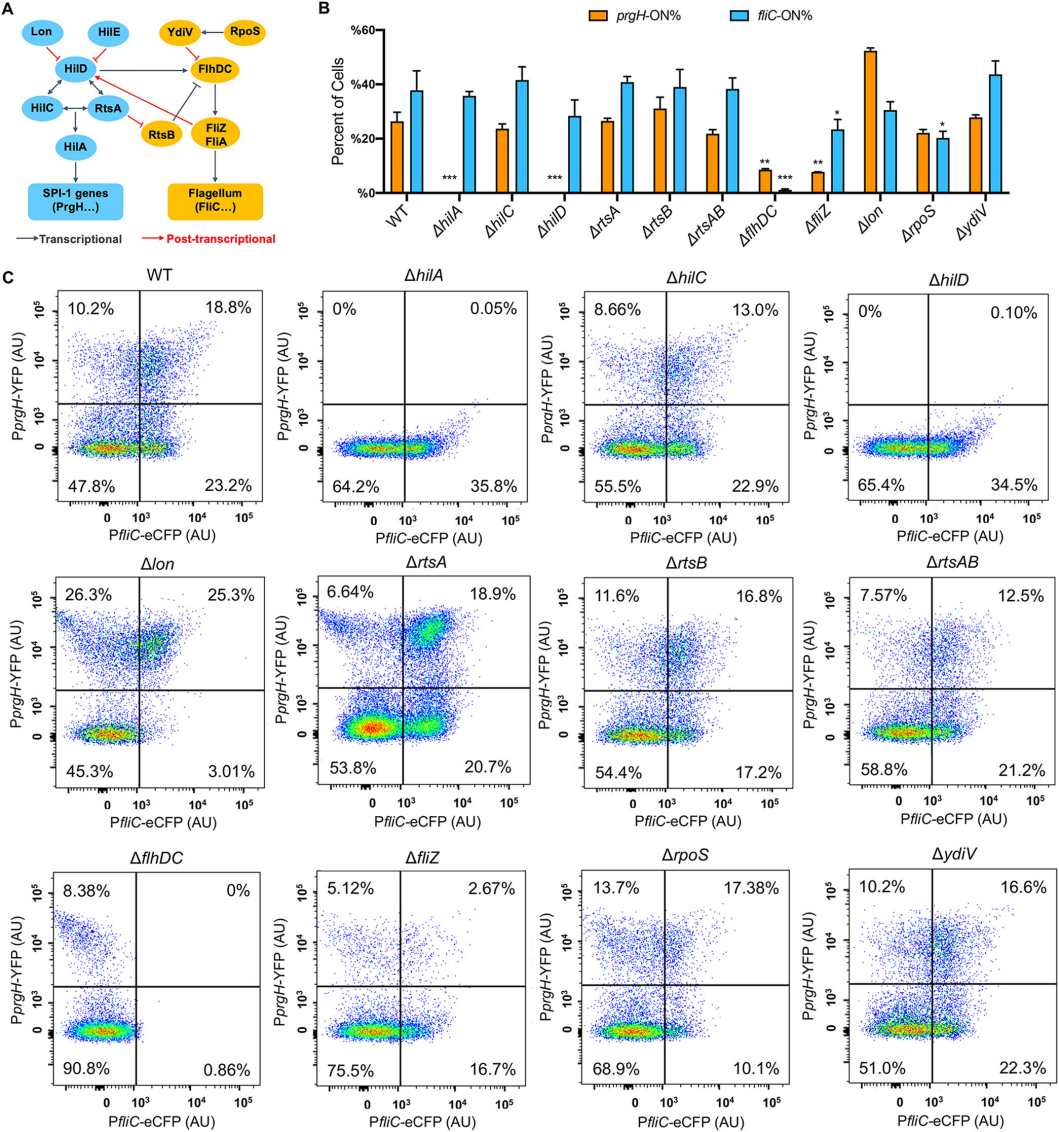
Cross talk between SPI-1 and flagellar pathways in single Salmonella cells. (A) Scheme of cross talk between SPI-1 and flagellar genes. (B and C) Flow cytometry analysis of *prgH* and *fliC* expression in *S.* Typhimurium variants. The experimental procedure and data analysis are the same as those described in the legend of Fig. 1B. Error bars in panel B represent 1 SD from the mean (*n* = 3). The *P* values were determined using the unpaired *t* test compared with the WT. **, *P* < 0.01; ***, *P* < 0.001. The results in panel C are representative of data from three biological replicates.

Our recent study of the role of protein synthesis in Salmonella virulence suggests that perturbing translational fidelity results in the downregulation of both SPI-1 and flagellar genes (29), leading us to further investigate the interplay between these two pathways. Previous studies have shown that the expressions of SPI-1 and flagellar genes in Salmonella are correlated at the population level (28). To monitor these two pathways concurrently in single cells, we constructed a low-copy-number plasmid carrying three fluorescence proteins. The *mCherry* gene (encoding a red fluorescent protein) is under the control of a constitutive P*tet* promoter, serving as a reference for the overall gene expression activity in cells; the *YFP* gene (encoding a yellow fluorescent protein) is controlled by the promoter of *prgH*, which is a downstream SPI-1 gene; and the *eCFP* gene (encoding an enhanced cyan fluorescent protein) is fused to the promoter of *fliC*, a class 3 flagellar gene encoding the flagellin, as a reporter for the expression of the flagellar pathway. Using fluorescence microscopy, we observed the bimodal expression of both the *prgH* and *fliC* promoters in wild-type (WT) *S.* Typhimurium ATCC 14028s cells grown in high-salt Luria broth (LB Miller) (Fig. 1A). This was consistent with previous results from single reporters of SPI-1 and flagellar pathways, respectively (24, 25). Further flow cytometry analysis revealed that the expression of *prgH* and *fliC* was indeed positively correlated (Fig. 1B to D; see also Fig. S1 in the supplemental material). The master regulator of SPI-1 genes is HilD, which controls the expression of HilA (28, 30, 31). HilA in turn activates the expression of downstream SPI-1 genes, including *prgH* (Fig. 2A). As expected, deleting *hilD* or *hilA* abolished the expression of the *prgH* promoter, whereas deleting the master flagellar regulator *flhDC* abolished the expression of P*fliC* (Fig. 2B and C). These results confirm that our triple-fluorescence reporter accurately and sensitively measures the expression of both the SPI-1 and flagellar pathways in single cells.

10.1128/mBio.02374-21.1 FIG S1 Flow cytometry of WT Salmonella. (A and B) WT *S.* Typhimurium carrying either the control plasmid pZS P*tet-lacZ* without fluorescence (A) or pZS P*tet*-*mCherry* P*prgH-YFP* P*fliC-eCFP* (B). (C) Time course of P*fliC*-eCFP distribution using WT *S.* Typhimurium carrying pZS P*tet*-*mCherry* P*prgH-YFP* P*fliC-eCFP*. The sample preparation is the same as the one described in the legend of Fig. 1C. Download FIG S1, PDF file, 1.6 MB. Copyright © 2021 Lyu et al. 2021 Lyu et al. https://creativecommons.org/licenses/by/4.0/ This content is distributed under the terms of the Creative Commons Attribution 4.0 International license.

We also found that deleting *flhDC* or *fliZ* decreased the fraction of *prgH-*ON cells (Fig. 2), supporting the cross talk between flagellar and SPI-1 pathways at the single-cell level.

**FIG 3:**
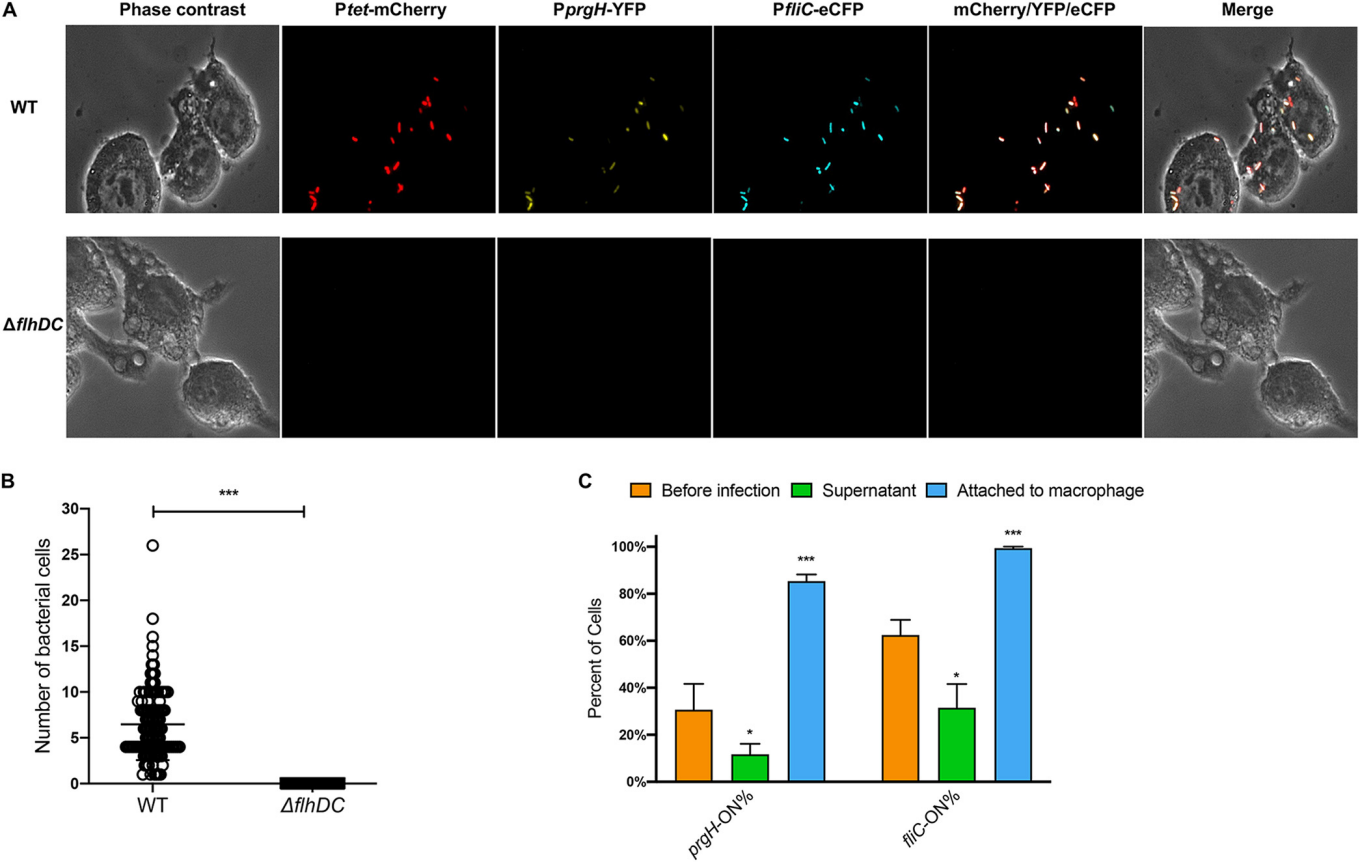
SPI-1 and flagellar pathways are critical for macrophage attachment. WT and Δ*flhDC S.* Typhimurium cells carrying pZS P*tet*-*mCherry* P*prgH-YFP* P*fliC-eCFP* were grown in LB Miller at 37°C for 5 h to the early stationary phase and incubated with macrophage cells at an MOI of 50 for 15 min without centrifugation. The cultures were then washed to remove Salmonella cells that were not attached to macrophages. (A) Phase-contrast and fluorescence microscopy of macrophages with Salmonella cells. Most Salmonella cells attached to macrophages were *prgH*-ON and *fliC-*ON. The images are representative of results from four biological replicates. (B) Quantitation of Salmonella cells attached to individual macrophages. Each dot represents a macrophage cell. The data are combined from four replicates. (C) Quantitation of the percentages of *prgH*-ON and *fliC-*ON cells in the Salmonella populations before infection, within the supernatant postinfection, and attached to macrophages. Error bars represent 1 SD from the mean (*n* = 4). The *P* value was determined using the unpaired *t* test. *, *P* < 0.05; ***, *P* < 0.001.

Previous studies suggest that nonmotile Salmonella mutants are defective in host invasion (32). We confirmed that deleting *flhDC* indeed abolished the attachment of Salmonella to macrophage cells (Fig. 3). Interestingly, in the WT strain, *prgH-*ON/*fliC*-ON cells were substantially enriched in the subpopulation that attached to macrophages (Fig. 3), suggesting that both SPI-1 and flagellar pathways are required for effective host cell interactions.

### Flagellar expression decreases the tolerance of single Salmonella cells to antibiotics.

**FIG 4:**
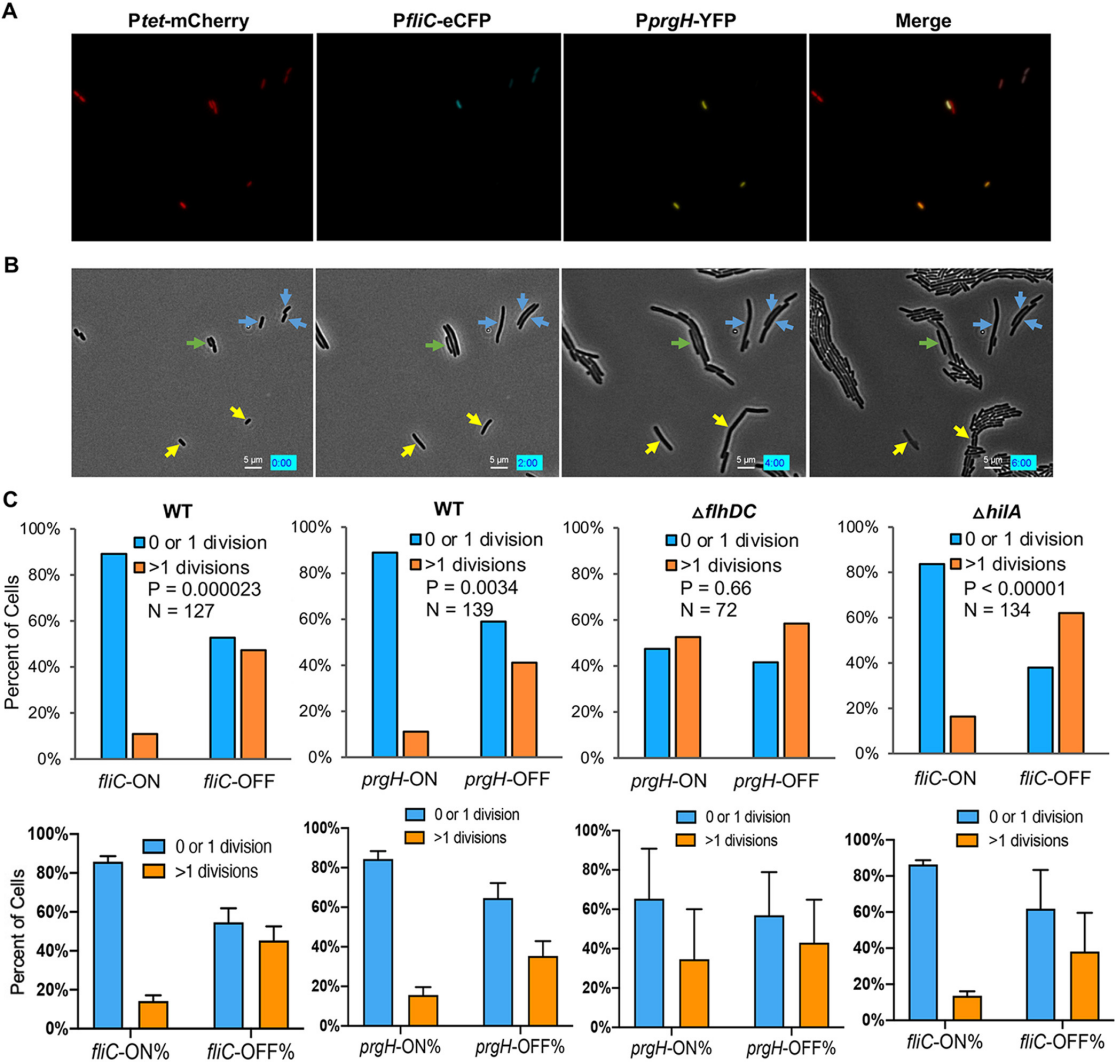
Increased tolerance to Cipro in *fliC*-OFF Salmonella cells. (A) Fluorescence microscopy of WT *S.* Typhimurium carrying pZS P*tet*-*mCherry* P*prgH-YFP* P*fliC-eCFP*. Cells were grown in LB Miller at 37°C for 5 h to the early stationary phase, treated with 0.1 μg/ml (7 times the MIC) Cipro for 15 min, and spread onto an LB agar pad prior to microscopy. (B) Time-lapse growth of cells in panel A. Yellow, cyan, and green arrows indicate *prgH-*ON/*fliC-*OFF, *prgH-*OFF/*fliC-*ON, and *prgH-*ON/*fliC-*ON cells, respectively. The other cells are *prgH*-OFF/*fliC*-OFF. While some cells resumed multiple divisions, others underwent no division or only one division before growth arrest or cell lysis. (C) Statistics of *fliC-*ON and *fliC-*OFF cells surviving Cipro treatment in panels A and B. *fliC*-OFF cells show a significantly higher level of survival following Cipro treatment than *fliC*-ON cells in the WT and Δ*hilA* strains. These results are representative (top) and averages (bottom) of data from at least two independent experiments. The *P* values were determined using the χ^2^ test.

**FIG 5:**
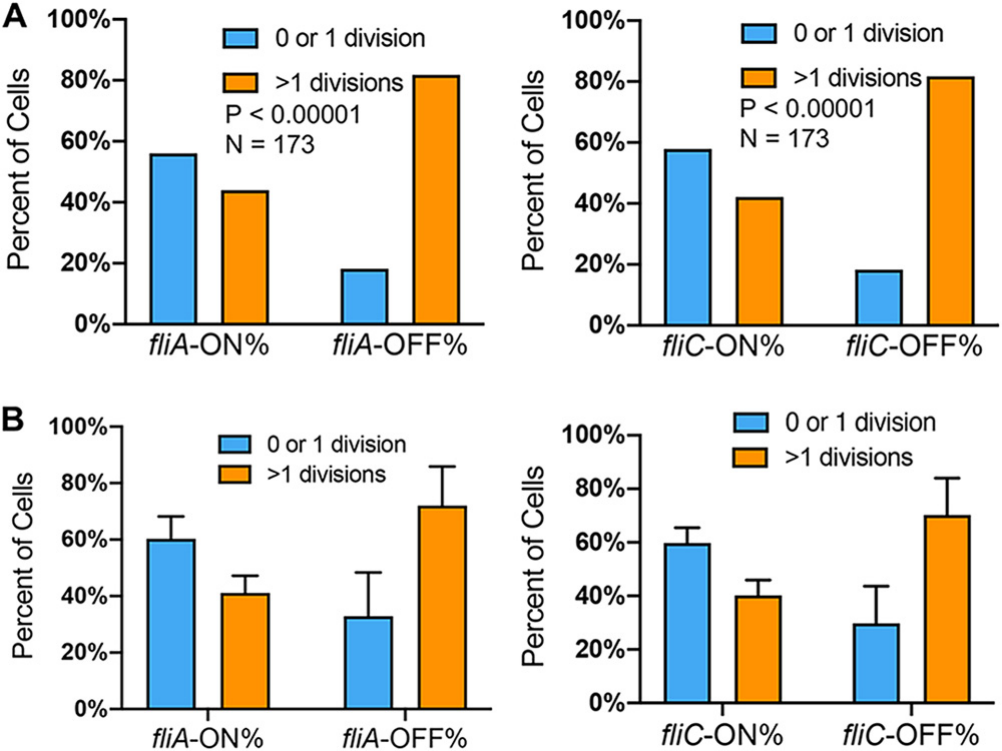
Increased tolerance to Cipro in *fliA*-OFF cells. WT *S*. Typhimurium cells carrying pZS P*tet*-*mCherry* P*fliA-YFP* P*fliC-eCFP* were grown and treated as described in the legend of Fig. 3. These results are representative (top) and averages (bottom) of data from three independent experiments. The *P* values were determined using the χ^2^ test.

We next investigated the phenotypic variations of *fliC*-ON and *fliC-*OFF cells. We recorded the motility of single Salmonella cells using fluorescence and phase-contrast microscopy. While ∼50% of *fliC*-ON cells were highly motile, only <10% of *fliC-*OFF cells showed motility (Fig. S2 and Video S1). In addition to FliC, some Salmonella cells also encode another flagellin, FljB, in phase variation (33). The small percentage of motile cells among the *fliC-*OFF cells could be due to FljB-dependent motility. Next, we tested the regrowth of early-stationary-phase cells and did not observe a significant difference between the *fliC*-ON and *fliC-*OFF subpopulations (Fig. S3). However, following a brief treatment (15 min) of early-stationary-phase Salmonella cells with the bactericidal antibiotic ciprofloxacin (Cipro) or streptomycin (Strep), a significantly higher percentage of *fliC-*OFF cells resumed multiple divisions than *fliC*-ON cells (Fig. 4, Fig. S4, and Video S2). The expression of flagellar genes is multilayered, and class 3 genes (such as *fliC*) are under the control of the sigma factor FliA. We further validated that similar to *fliC-*OFF cells, *fliA-*OFF cells were also more tolerant to antibiotic killing than *fliA-*ON cells (Fig. 5).

10.1128/mBio.02374-21.2 FIG S2 Motility of single Salmonella cells with *fliC-*ON or *fliC-*OFF. Early-stationary-phase cells carrying pZS P*fliC-eCFP* and grown in LB Miller at 37°C were spotted onto a microscope slide and visualized using phase-contrast and fluorescence microscopy. (A) Phase-contrast and fluorescence images taken immediately before the video. Red and cyan ovals indicate *fliC-*OFF and *fliC-*ON cells, respectively. Note that some *fliC*-ON cells are highly motile (e.g., 8 and 10) and shift positions between phase-contrast and fluorescence images within seconds. Some motile cells are not captured in both images. (B) Quantitation of the results reveals that a significantly large fraction of *fliC-*ON cells are motile compared with *fliC-*OFF cells. Some nonmotile *fliC-*ON cells might result from incorrect assembly or a loss of the flagella during growth and treatment. These results are representative (left) and averages (right) of data from three independent experiments. The *P* value was determined using the χ^2^ test. Download FIG S2, PDF file, 0.4 MB. Copyright © 2021 Lyu et al. 2021 Lyu et al. https://creativecommons.org/licenses/by/4.0/ This content is distributed under the terms of the Creative Commons Attribution 4.0 International license.

10.1128/mBio.02374-21.3 FIG S3 Regrowth of early-stationary-phase cells without antibiotic treatment. WT Salmonella cells carrying pZS P*tet*-*mCherry* P*prgH-YFP* P*fliC-eCFP* were grown in LB Miller at 37°C to the early stationary phase, spread onto an LB agar pad, and subjected to time-lapse microscopy. (A) Fluorescence and phase-contrast time-lapse images. Cyan and green arrows indicate *prgH-*OFF/*fliC-*ON and *prgH-*ON/*fliC-*ON cells, respectively. The other cells are *prgH*-OFF/*fliC*-OFF. (B) Histograms of the time that it takes cells to reach the first division. These results are representative (top) and averages (bottom) of data from four independent experiments. Download FIG S3, PDF file, 0.5 MB. Copyright © 2021 Lyu et al. 2021 Lyu et al. https://creativecommons.org/licenses/by/4.0/ This content is distributed under the terms of the Creative Commons Attribution 4.0 International license.

10.1128/mBio.02374-21.4 FIG S4 *fliC*-OFF cells exhibit increased tolerance to streptomycin (Strep). WT *S.* Typhimurium cells carrying pZS P*tet*-*mCherry* P*prgH-YFP* P*fliC-eCFP* were grown in LB Miller at 37°C for 5 h to the early-stationary phase, treated with 60 μg/ml Strep for 15 min, and spread onto an LB agar pad prior to time-lapse microscopy. These results are representative (left) and averages (right) of data from two independent experiments. The *P* values were determined using the χ^2^ test. Download FIG S4, PDF file, 0.1 MB. Copyright © 2021 Lyu et al. 2021 Lyu et al. https://creativecommons.org/licenses/by/4.0/ This content is distributed under the terms of the Creative Commons Attribution 4.0 International license.

10.1128/mBio.02374-21.9 VIDEO S1 Motility of *fliC-*OFF and *fliC-*ON cells (related to Fig. S2 in the supplemental material). Download Movie S1, MOV file, 0.4 MB. Copyright © 2021 Lyu et al. 2021 Lyu et al. https://creativecommons.org/licenses/by/4.0/ This content is distributed under the terms of the Creative Commons Attribution 4.0 International license.

10.1128/mBio.02374-21.10 VIDEO S2 Growth of WT Salmonella with Cipro treatment (related to Fig. 4). Download Movie S2, MOV file, 1.1 MB. Copyright © 2021 Lyu et al. 2021 Lyu et al. https://creativecommons.org/licenses/by/4.0/ This content is distributed under the terms of the Creative Commons Attribution 4.0 International license.

Early-stationary-phase *prgH-*OFF cells also appeared to survive Cipro treatment better than *prgH-*ON cells. Given the cross talk between flagellar and SPI-1 genes, we next tested which pathway was primarily responsible for the variation in antibiotic tolerance. We found that deleting *flhDC* abolished the difference in Cipro tolerance between *prgH-*ON and *prgH-*OFF cells, whereas *fliC-*OFF cells remained more tolerant than *fliC*-ON cells in the Δ*hilA* strain (Fig. 4C). Deleting *flhDC* also increased the fraction of Cipro-tolerant cells compared with the WT (Fig. S5A). Collectively, these results suggest that heterogeneous flagellar expression, instead of the SPI-1 pathway, promotes variation in transient antibiotic tolerance in Salmonella under the tested conditions.

10.1128/mBio.02374-21.5 FIG S5 Deleting flagella increases tolerance to Cipro. Cells were treated as described in the legend of Fig. 3. (A) Deleting *flhDC* increases survival upon Cipro treatment. (B) Deleting *motAB* abolishes the difference of Cipro sensitivity between *fliC-*ON and *fliC-*OFF cells. These results are representative (top) and averages (bottom) of data from three independent experiments. The *P* values were determined using the χ^2^ test. Download FIG S5, PDF file, 0.2 MB. Copyright © 2021 Lyu et al. 2021 Lyu et al. https://creativecommons.org/licenses/by/4.0/ This content is distributed under the terms of the Creative Commons Attribution 4.0 International license.

### Flagella compete with efflux for PMF to decrease antibiotic tolerance.

**FIG 6:**
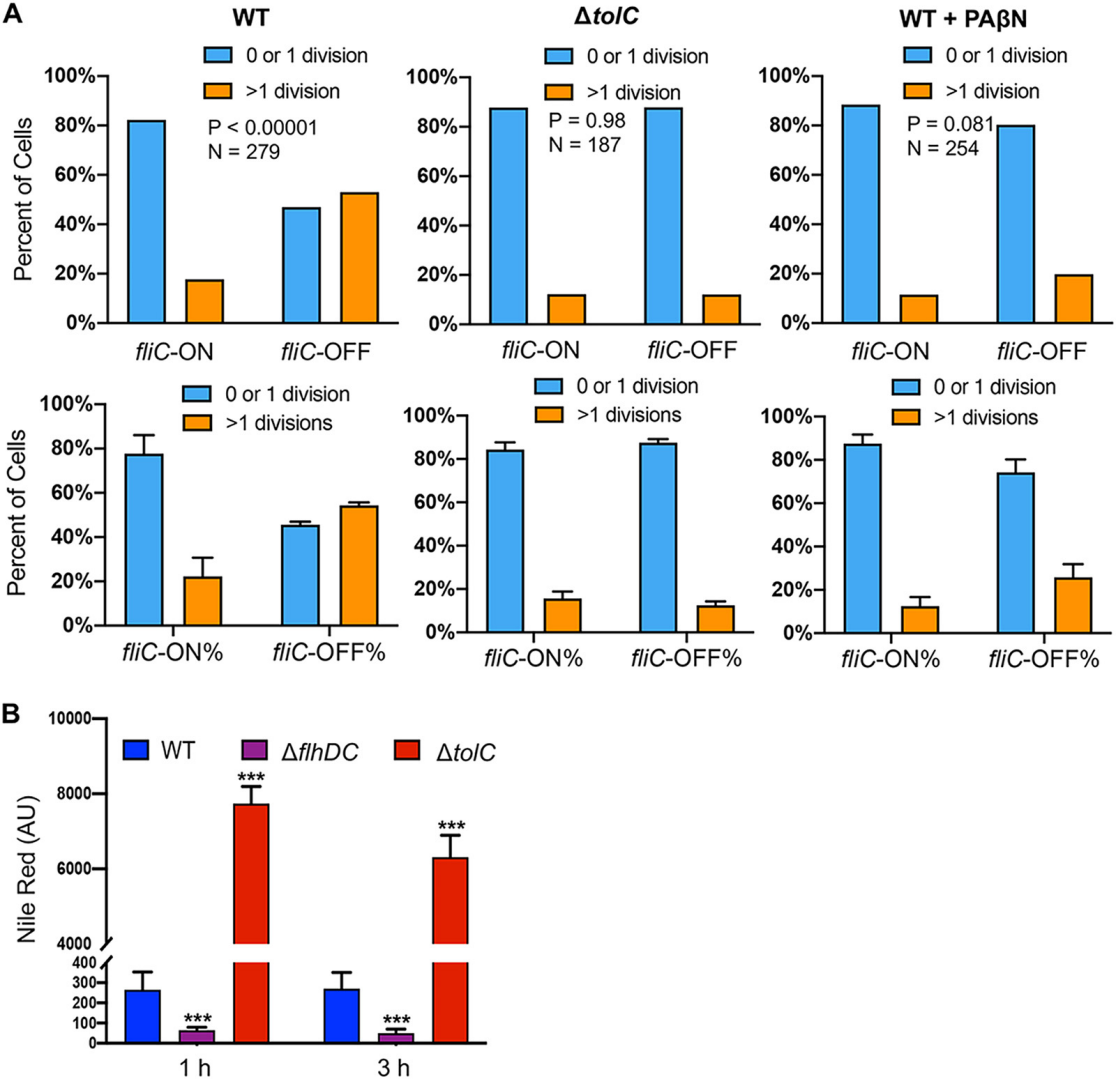
Increased antibiotic tolerance in *fliC*-OFF cells depends on efflux. (A) Statistics of *fliC-*ON and *fliC-*OFF *S*. Typhimurium cells surviving Cipro (0.1 μg/ml) treatment as described in the legend of Fig. 3. Cells in the right panels were treated with the efflux inhibitor PAβN (20 μg/ml) together with Cipro. These results are representative (top) and averages (bottom) of data from three independent experiments. The *P* values were determined using the χ^2^ test. (B) *S.* Typhimurium cells grown to the early stationary phase were treated with the efflux reporter dye Nile red (48 μg/ml) and monitored for fluorescence using a plate reader. As expected, due to the efflux defect, the Δ*tolC* mutant accumulated more Nile red than the WT. The Δ*flhDC* mutant displayed a significantly lower Nile red signal than the WT, indicating higher efflux activity. Error bars represent 1 SD from the mean (*n* = 4). The *P* value was determined using the unpaired *t* test. ***, *P* < 0.001.

**FIG 7:**
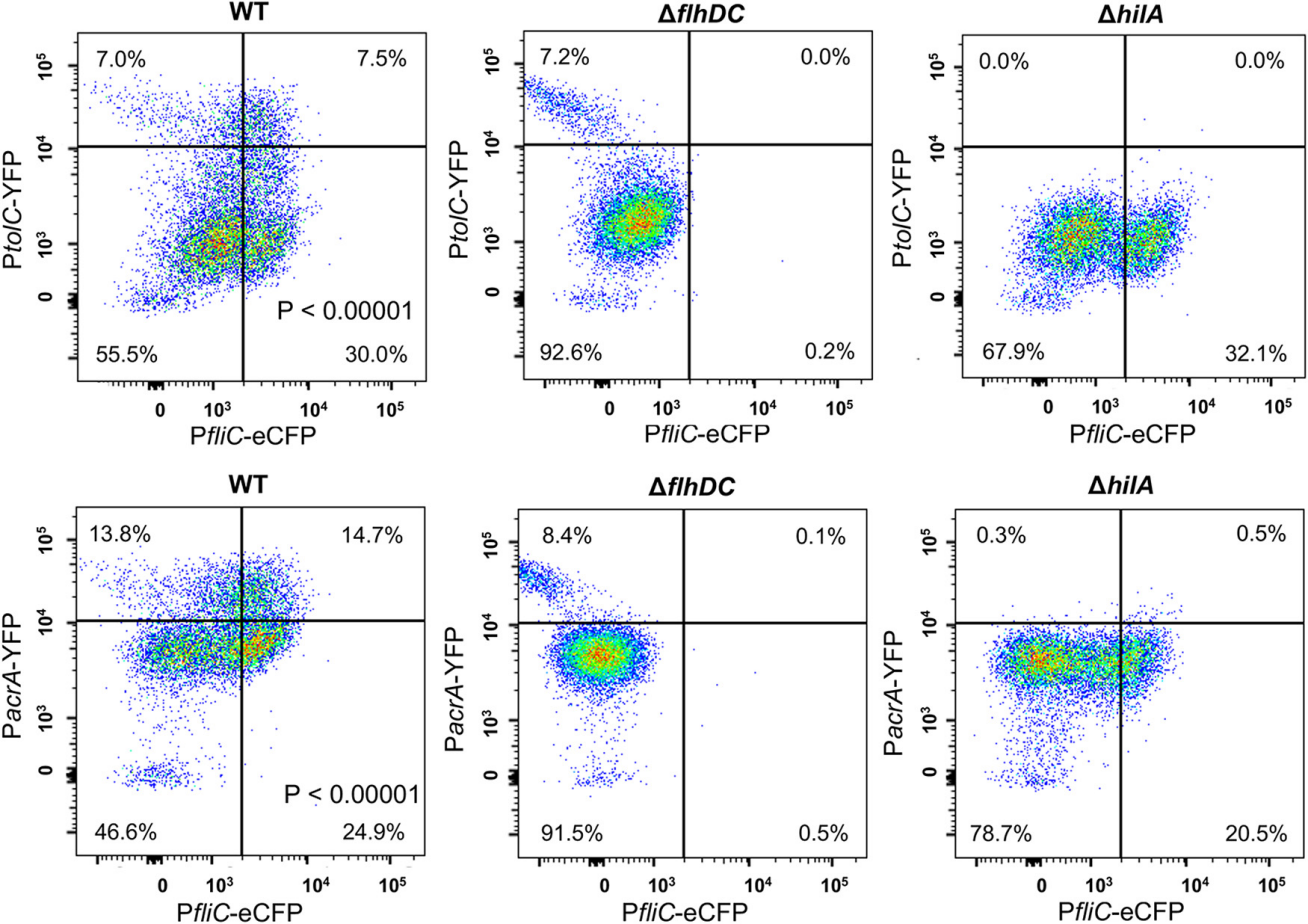
Flow cytometry of Salmonella variants carrying flagellar and efflux reporters. Salmonella variants carrying pZS P*tet*-*mCherry* P*tolC-YFP* P*fliC-eCFP* or pZS P*tet*-*mCherry* P*acrA-YFP* P*fliC-eCFP* were grown in LB Miller at 37°C for 5 h to the early stationary phase and analyzed by flow cytometry. AcrAB-TolC is a major multidrug efflux pump that removes Cipro and other antibiotics from Salmonella cells using proton motive force. Compared with *fliC*-OFF cells, *fliC*-ON cells in the WT strain exhibit high expression levels of P*tolC* and P*acrA*. Deleting *flhDC* or *hilA* also decreases the fractions of *tolC-*HIGH and *acrA-*HIGH cells. The *P* values were determined using the χ^2^ test. These results are representative of data from at least four biological replicates.

**FIG 8:**
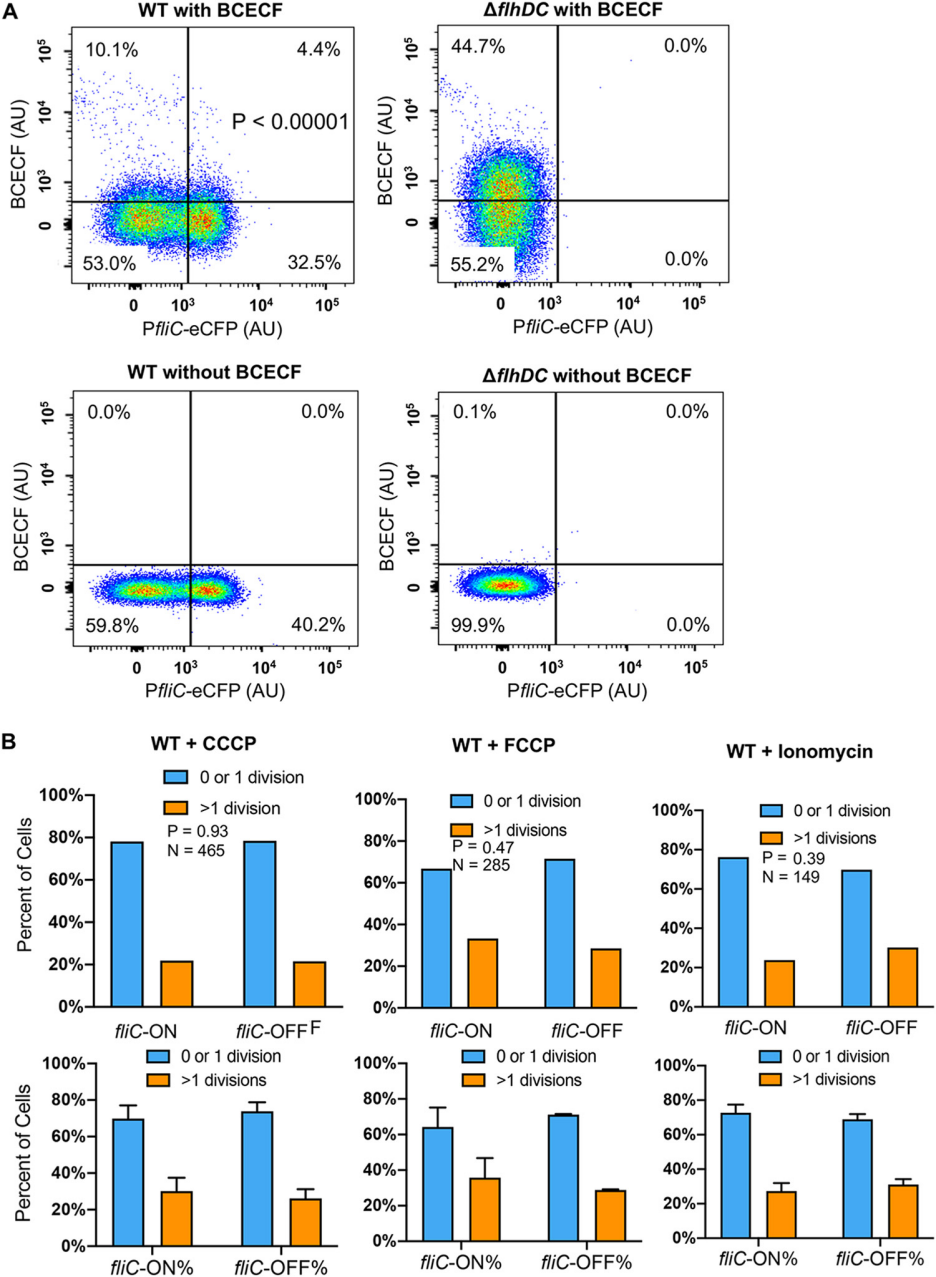
Proton motive force affects differential antibiotic tolerance between *fliC-*ON and *fliC-*OFF cells. (A) WT and Δ*flhDC* Salmonella strains carrying pZS P*fliC-eCFP* were grown to the early stationary phase, treated with the intracellular pH dye BCECF-AM (40 μM), and analyzed using flow cytometry. A significantly higher percentage of *fliC-*OFF cells have high BCECF signals (indicating higher pH and lower intracellular protein concentrations) than *fliC*-ON cells. Deleting *flhDC* also increases the fraction of high-BCECF cells. The *P* value was determined using the χ^2^ test (*n* = ∼30,000). These results are representative of data from at least three biological replicates. (B) Statistics of *fliC-*ON and *fliC-*OFF *S*. Typhimurium cells surviving Cipro (0.1 μg/ml) treatment as described in the legend of Fig. 3. The addition of the ionophore CCCP (10 μg/ml), FCCP (10 μg/ml), or ionomycin (1 μg/ml) abolished the difference in Cipro tolerance between *fliC-*ON and *fliC-*OFF cells. CCCP and FCCP are proton ionophores; ionomycin causes the efflux of cations like Ca^2+^ and the simultaneous influx of protons (54). Therefore, all three ionophores disrupt the PMF. These results are representative (top) and averages (bottom) of data from three independent experiments. The *P* values were determined using the χ^2^ test.

**FIG 9:**
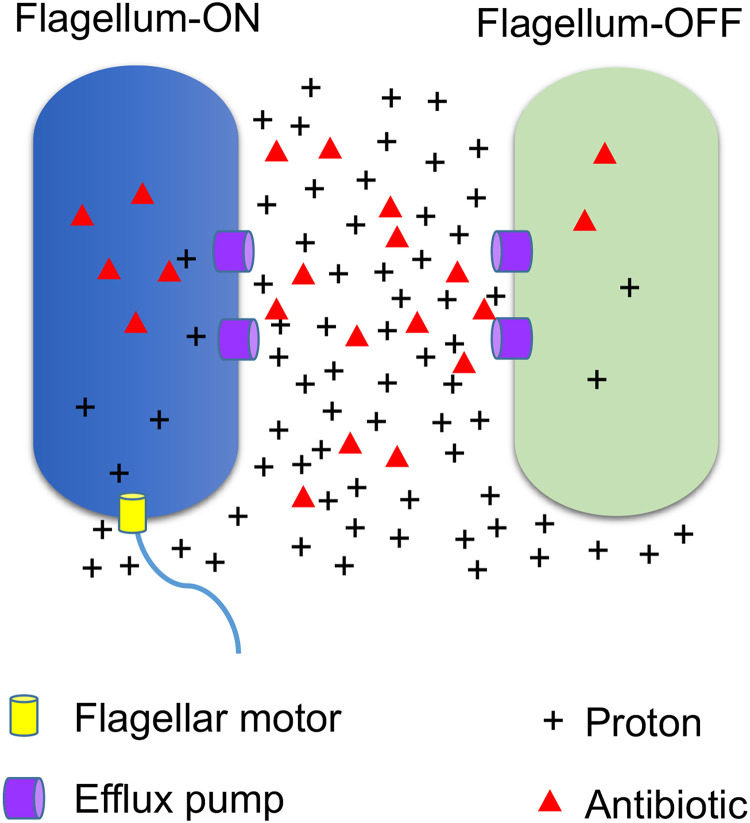
Model for the trade-off between flagellar expression and efflux activity leading to antibiotic tolerance. Flagellar rotation and efflux are two of the major biological processes that use PMF as the driving force. Motile cells (flagellum-ON) consume PMF through the continuous rotation of flagella, which decreases the PMF and, therefore, the efflux activity. This leads to the accumulation of a higher level of intracellular toxic molecules, including antibiotics, in flagellum-ON cells than in flagellum-OFF cells.

Many Gram-negative bacteria, including Salmonella, exhibit robust efflux activities to remove toxic small molecules (e.g., antibiotics) from cells (34, 35). TolC is a key component for many resistance-nodulation-cell division (RND) efflux pumps, which use PMF to move antibiotics (including Cipro and Strep) across the double membrane from the cytoplasm to the exterior (34–37). We showed that deleting *tolC* or adding an efflux pump inhibitor, Phe-Arg-β-naphthylamide (PAβN) (38), abolished the difference between *fliC-*ON and *fliC-*OFF cells in Cipro tolerance (Fig. 6A). Using an efflux reporter dye, Nile red (39), we found that the Δ*flhDC* mutant exhibited significantly higher efflux activity than the WT (Fig. 6B). In contrast, deleting *hilA* or *hilD* only modestly affected efflux (Fig. S6). This could not be explained by the expression of efflux genes, as deleting *flhDC* appeared to decrease, rather than increase, the percentage of cells with high expression levels of *tolC* (*tolC-*HIGH) or *acrAB* (*acrAB*-HIGH) (Fig. 7). In the WT, the *fliC*-ON subpopulation also displayed higher percentages of *tolC-*HIGH and *acrAB*-HIGH cells (Fig. 7). As both efflux and flagellar motility are driven by PMF, we hypothesized that there is a trade-off between efflux and flagellar expression due to the competition for PMF. Indeed, adding the PMF uncoupler carbonyl cyanide *m*-chlorophenylhydrazone (CCCP) or carbonyl cyanide *p*-trifluoromethoxyphenylhydrazone (FCCP) ablated the difference observed between *fliC-*ON and *fliC-*OFF cells in Cipro tolerance (Fig. 8). Deleting the *motAB* genes, which encode flagellar motor proteins that drive the rotation of flagella using PMF, also abolished the difference in antibiotic tolerance between *fliC-*ON and *fliC-*OFF cells (Fig. S5B). Furthermore, we used an intracellular pH indicator, BCECF-AM [2′,7′-bis-(2-carboxyethyl)-5 (and -6)-carboxyfluorescein acetoxymethyl ester], and found that in *fliC*-OFF cells, a larger percentage exhibited lower intracellular proton concentrations (higher BCECF signals and pH) than in *fliC-*ON cells (Fig. 8). The Δ*flhDC* mutant also had a larger percentage of cells with higher BCECF signals than the WT. As the extracellular pH remained the same for all cells, this implies that *fliC-*OFF cells have higher ΔpH (and likely higher PMF) than *fliC-*ON cells overall. Furthermore, the addition of glucose to the media abolished the difference in Cipro tolerance between *fliC-*ON and *fliC-*OFF cells (Fig. S7), presumably due to an increase in PMF. Together, our data support that the expression of flagella costs PMF and reduces efflux efficiency, thereby decreasing tolerance to antibiotics (Fig. 9).

10.1128/mBio.02374-21.6 FIG S6 Effect of SPI-1 deletion on efflux. The Nile red efflux assay was performed as described in the legend of Fig. 6B. Error bars represent 1 SD from the mean (*n* = 12). The *P* value was determined using the unpaired *t* test. *, *P* < 0.05; **, *P* < 0.01. Download FIG S6, PDF file, 0.1 MB. Copyright © 2021 Lyu et al. 2021 Lyu et al. https://creativecommons.org/licenses/by/4.0/ This content is distributed under the terms of the Creative Commons Attribution 4.0 International license.

10.1128/mBio.02374-21.7 FIG S7 The addition of glucose abolishes the difference in Cipro tolerance between *fliC-*ON and *fliC*-OFF cells. The experiments were performed as described in the legend of Fig. 4, except that 1% glucose was added to the culture 30 min before Cipro treatment. These results are representative (top) and averages (bottom) of data from at least two independent experiments. The *P* values were determined using the χ^2^ test. Download FIG S7, PDF file, 0.2 MB. Copyright © 2021 Lyu et al. 2021 Lyu et al. https://creativecommons.org/licenses/by/4.0/ This content is distributed under the terms of the Creative Commons Attribution 4.0 International license.

## DISCUSSION

Flagellar motility is a mechanism commonly used by bacteria to search for nutrients and hosts. However, the expression and assembly of flagella are also energetically costly processes (40, 41). It is remarkable that some bacteria, such as Salmonella, use a bet-hedging mechanism to express flagellar genes heterogeneously within a population (23, 24, 42). Our work here reveals a previously unknown benefit of the differential expression of flagellar genes. We show that *fliC-*OFF Salmonella cells that do not express flagella are more tolerant to antibiotics (Fig. 4; see also Fig. S4 in the supplemental material). Due to the cost of proton motive force to drive motility, flagellum-ON cells are less capable of removing intracellular antibiotics through efflux than flagellum-OFF cells (Fig. 9). It is also interesting to note that the expression of flagellar and SPI-1 genes appears to be positively correlated (Fig. 1). SPI-1 genes are essential for Salmonella to invade host cells (15), and nonmotile Salmonella mutants have been shown to exhibit a substantial decrease in host cell invasion *in vitro* and *in vivo* (32). Consistently, we show that Δ*flhDC* mutant cells are defective in attachment to macrophage cells (Fig. 3). At the single-cell level, macrophage-attached Salmonella cells are significantly enriched in *prgH-*ON and *fliC-*ON cells (Fig. 3). Coupling the expression of the SPI-1 secretion system with flagella would therefore enable *prgH-*ON/*fliC*-ON cells to quickly move toward and invade the host cells, whereas *prgH-*OFF/*fliC-*OFF cells remain in the extracellular environment, such as the intestinal lumen of mammalian hosts. The intestinal lumen is enriched in antimicrobial molecules, such as antimicrobials secreted by competing microbes as well as bile acids from the host (43, 44). Shutting off both SPI-1 and flagellar pathways would enable these Salmonella cells to maximize their efflux activity and remove toxic molecules, allowing survival and growth.

The cross talk between SPI-1 and flagellar pathways is complex and involves multiple factors, including FlhDC, FliZ, RtsAB, and HilD. HilD, RtsA, and HilC form a positive feedforward loop to control the expression of SPI-1 genes (45). A previous study shows that overexpressing HilD activates the transcription of *flhDC* (46). Our reporter assay reveals that deleting *hilD* does not substantially decrease the fraction of *fliC-*ON cells (Fig. 2), suggesting that the native expression level of HilD under our growth conditions may not be high enough to dominate the cross talk between the two pathways. We also show that deleting *flhDC* or *fliZ* negatively impacts both the SPI-1 and flagellar pathways (Fig. 2), which is consistent with previous studies (47, 48).

The hierarchical expression of flagellar genes is regulated at multiple levels (49). The class 1 master regulator FlhDC controls the expression of class 2 genes such as *fliA*, and FliA controls the expression of class 3 genes. Salmonella encodes two class 3 flagellins, FliC and FljB, and undergoes phase variation (33). Our motility assay shows that the fraction of motile cells is 7-fold higher in the *fliC-*ON group than in the *fliC-*OFF group (Fig. S2), suggesting that FliC is the prevalent form of flagellin under our tested conditions. The small percentage of motile cells among the *fliC-*OFF cells could be due to FljB-dependent motility. To test the effect of phase variation on the heterogeneity of antibiotic tolerance, we constructed a reporter using YFP under the control of P*fliA*, which is upstream of phase variation. We show that like *fliC-*ON cells, *fliA-*ON cells are also more sensitive to antibiotic killing than *fliA-*OFF cells (Fig. 5), further supporting our model of a flagellum-efflux trade-off (Fig. 9).

Antibiotic tolerance and resistance pose a severe and urgent threat to human health (6–8). A significant cause of antibiotic failure is the efflux activity in many bacterial pathogens, especially the Gram-negative bacteria (34). Salmonella expresses multiple efflux pumps. One major family is the TolC-dependent RND efflux pumps, which use PMF to remove many clinically important antibiotics, such as Cipro and Strep used in this study (34–37). Cipro and Strep are representatives of fluoroquinolone and aminoglycoside antibiotics that target DNA replication and protein synthesis, respectively, and Cipro is frequently used as a frontline antibiotic to treat Salmonella infections. We show that the differential tolerance to Cipro between *fliC-*ON and *fliC*-OFF cells depends on the presence of TolC and PMF (Fig. 5 to 7), revealing a novel mechanism of antibiotic tolerance resulting from the trade-off between flagellar motility and efflux. It is therefore advantageous for the Salmonella population to maintain the heterogeneous expression of flagellar genes among individual cells, which provides a bet-hedging mechanism for optimal adaptation to ever-changing environments. In addition to Salmonella, heterogeneous expression of flagella has been observed in some pathogenic Escherichia coli strains (50). Given that PMF is used to drive flagellar motility and efflux in many bacteria (35, 41), it is tempting to speculate that the trade-off between the two also occurs in other bacterial species, which needs to be investigated in future studies. Previous studies have also suggested that the heterogeneous expression of SPI-1 cells contributes to persistence in the Salmonella population (51, 52). Our work here shows that the flagellar pathway, instead of SPI-1, contributes to the transient tolerance to antibiotics (Fig. 3), thus highlighting distinct mechanisms underlying antibiotic tolerance and persistence.

## MATERIALS AND METHODS

### Bacterial strains, plasmids, and growth conditions.

All Salmonella strains (Table 1) used in this study are derived from *S*. Typhimurium ATCC 14028s. Salmonella serovar Typhimurium gene deletion mutants were constructed essentially as previously described (53). Briefly, the Flp recombination target (FRT)-flanked chloramphenicol resistance gene (*cat*) was amplified by PCR from plasmid pKD3 using primers shown in Table S1 in the supplemental material. The resulting PCR products were purified and electroporated into *S*. Typhimurium ATCC 14028s cells harboring plasmid pKD46 expressing the Red recombinase. The recombinants were selected on Luria broth (LB) plates containing chloramphenicol at 37°C and verified by PCR. All strains used in this study were cultured in LB Lennox (containing 10 g/liter of tryptone, 5 g/liter of yeast extract, and 5 g/liter of NaCl) or LB Miller (containing 10 g/liter of tryptone, 5 g/liter of yeast extract, and 10 g/liter of NaCl). The following antibiotics were used: ciprofloxacin at 0.1 μg/ml, ampicillin at 100 μg/ml, chloramphenicol at 25 μg/ml, and streptomycin at 60 μg/ml.

**TABLE 1:** List of strains and plasmids^*a*^

Strain, plasmid, chemical, peptide, recombinant protein, or assay	Source or reference	Description or origin
Strains		
*S.* Typhimurium ATCC 14028s (WT)	ATCC	NA
Δ*hilA* (Δ*hilA*::*cat*)	This study	Region from positions 3040096–3041751 (Δ2–552 aa) replaced by *cat*
Δ*hilC* (Δ*hilC*::*cat*)	This study	Region from positions 3032347–3033228 (Δ2–294 aa) replaced by *cat*
Δ*hilD* (Δ*hilD*::*cat*)	Lab collection	Region from positions 3038076–3038999 (Δ2–308 aa) replaced by *cat*
Δ*lon* (Δ*lon*::*cat*)	Lab collection	Region from positions 506238–508586 (Δ2–784 aa) replaced by *cat*
Δ*rtsA* (Δ*rtsA*::FRT)	This study	Region from positions 4573934–4574584 (Δ2–217 aa) replaced by FRT
Δ*rtsB* (Δ*rtsB*::*cat*)	This study	Region from positions 4573428–4573715 (Δ1–96 aa) replaced by *cat*
Δ*rtsAB* (Δ*rtsAB*::*cat*)	This study	Region from positions 4573428–4574584 (Δ*rtsA*2–292 aa Δ*rtsB*1–96 aa) replaced by *cat*
Δ*flhDC* (Δ*flhDC*::*cat*)	This study	Region of positions 2032540–2033471 (Δ*flhD*1–117 aa Δ*flhC*1–193 aa) replaced by *cat*
Δ*fliZ* (Δ*fliZ*::*cat*)	Lab collection	Region from positions 2055542–2056093 (Δ2–183 aa) replaced by *cat*
Δ*rpoS* (Δ*rpoS*::*cat*)	Lab collection	Region from positions 3085731–3086723 (Δ1–331 aa) replaced by *cat*
Δ*ydiV* (Δ*ydiV*::*cat*)	This study	Region from positions 1432777–1433484 (Δ2–237 aa) replaced by *cat*

Plasmids		
pKD46	Lab collection	Rep101; Amp^r^
pKD3	Lab collection	R6K γ ori; Amp^r^ and Cam^r^
pCP20	Lab collection	Rep101(Ts); Amp^r^ and Cam^r^
pZS P*tet-mCherry* P*prgH*-*YFP* P*fliC*-*eCFP*	This study	Rep101; Amp^r^
pZS P*tet-mCherry* P*fliA-YFP* P*fliC-eCFP*	This study	Rep101; Amp^r^
pZS P*fliC-eCFP*	This study	Rep101; Amp^r^
pZS P*tet-mCherry* P*tet-YFP*	Lab collection	Rep101; Amp^r^
pZS P*tet-eCFP*	Lab collection	Rep101; Amp^r^

Chemicals, peptides, and recombinant proteins		
Ciprofloxacin	Acros Organics	Catalog no. 85721331
Chloramphenicol	Sigma	Catalog no. C0378
Ampicillin	Fisher Scientific	Catalog no. BP1760-5
Streptomycin	Sigma	Catalog no. S6051
CCCP	Alfa Aesar	Catalog no. L06932
FCCP	Tocris Bioscience	Catalog no. 045310
Ionomycin	Alfa Aesar	Catalog no. AAJ62448MCR
BCECF-AM	Biotium	Catalog no. 51011
Nile red	Acros Organics	Catalog no. 10658904

Critical commercial assays		
*Taq* Red master mix	Apex	Catalog no. 42138B
Q5 Hot Start high-fidelity 2× master mix	NEB	Catalog no. M0494S
In-Fusion HD cloning plus	Takara Bio USA, Inc.	Catalog no. 638909

a NA, not applicable; aa, amino acids; NEB, New England BioLabs.

10.1128/mBio.02374-21.8 TABLE S1 Oligonucleotides used in this study. Download Table S1, XLSX file, 0.01 MB. Copyright © 2021 Lyu et al. 2021 Lyu et al. https://creativecommons.org/licenses/by/4.0/ This content is distributed under the terms of the Creative Commons Attribution 4.0 International license.

For the construction of the P*prgH-YFP*, P*fliA-YFP*, and P*fliC-eCFP* fusions, the promoter regions of *prgH*, *fliA*, and *fliC* containing sequences of 500 bp upstream of the start codons were amplified from strain ATCC 14028s genomic DNA by PCR. The DNA fragments were fused to plasmid pZS P*tet-mCherry* P*tet-YFP* or pZS P*tet-eCFP* using the In-Fusion HD cloning kit according to the manufacturer’s instructions.

### Time-lapse microscopy.

Cultures grown overnight in LB Lennox were diluted 1:200 in LB Miller and grown aerobically for 5 h at 37°C. All cultures were normalized to an optical density at 600 nm (OD_600_) of ∼0.1, and ciprofloxacin was added to a final concentration of 0.1 μg/ml. Following 15 min of incubation at 37°C with agitation, cultures were harvested by centrifugation and resuspended in 100 μl LB. Two microliters of the resulting cultures was placed on a 1.5% agarose LB pad within a Gene Frame (Thermo Fisher Scientific). Fluorescence images were taken at the initial time point for quantitation. Cells were then imaged for 6 to 10 h at room temperature with a 60× phase-contrast lens at 20-min intervals. Image analysis was performed using a BZ-X800 analyzer (Keyence).

### Efflux activity assay.

Cultures of bacteria grown overnight were diluted 1:200 into fresh LB Miller and incubated for 5 h at 37°C. All cultures were normalized to the same OD_600_, and aliquots (100 μl) were transferred to 96-well plates. Nile red was added to the cells at a final concentration of 48 μg/ml, and the cells were incubated with agitation for 3 h at 37°C. The fluorescence intensity was recorded in a Synergy H1 microplate reader (BioTek) using an excitation wavelength of 549 nm and an emission wavelength of 628 nm.

### Flow cytometry analysis.

Cultures grown overnight in LB Lennox were diluted 1:200 in LB Miller and grown aerobically for 5 h at 37°C. BCECF acetoxymethyl ester (BCECF-AM) was added to a final concentration of 40 μM, and the cultures were incubated for 30 min at 37°C. Cells were diluted in phosphate-buffered saline (PBS) and directly measured on a BD FACSCanto II flow cytometer at a low flow rate. In all, 10,000 to 30,000 gated events were acquired for each sample. The density plots show the distribution of promoter activities in individual cells as determined based on YFP and eCFP fluorescence. Data were further analyzed using FlowJo software.

### Susceptibility to ciprofloxacin.

The MIC of ciprofloxacin was determined in 96-well microtiter plates. Mid-log-phase cultures were added to LB medium containing 2-fold serial dilutions of ciprofloxacin with a final inoculum size of 10^5^ CFU/ml. Plates were incubated at 37°C with agitation for 18 h. The MIC was defined as the lowest concentration that completely inhibited visible growth.

### Assay for attachment to macrophage cells.

J774A.1 (ATCC TIB-67) macrophage cells (∼10^5^ cells per well) were seeded into 96-well glass-bottom plates and left to adhere for 18 h. Infection was conducted by adding early-stationary-phase (∼5 h) bacterial cells to each well at a multiplicity of infection (MOI) of 50. The plates were incubated at 37°C in a CO_2_ incubator for 15 min without centrifugation. Nonadherent bacteria were then removed by three washes with PBS. The macrophage cells were fixed with 4% paraformaldehyde (PFA) for 10 min and immediately imaged with a Keyence BZ-X800 fluorescence microscope.

### Statistical analyses.

Experiments were performed using at least three biological replicates. In all cases, error bars represent the standard deviations (SD). Statistical differences were analyzed using the χ^2^ test or the unpaired *t* test.
