# Supplementary figures and images for: Heterogeneous Flagellar Expression in Single Salmonella Cells Promotes Diversity in Antibiotic Tolerance
Source: mBio. 2021 Sep 28;12(5):e02374-21. doi: 10.1128/mBio.02374-21 (PMC8546535; doi:10.1128/mBio.02374-21)

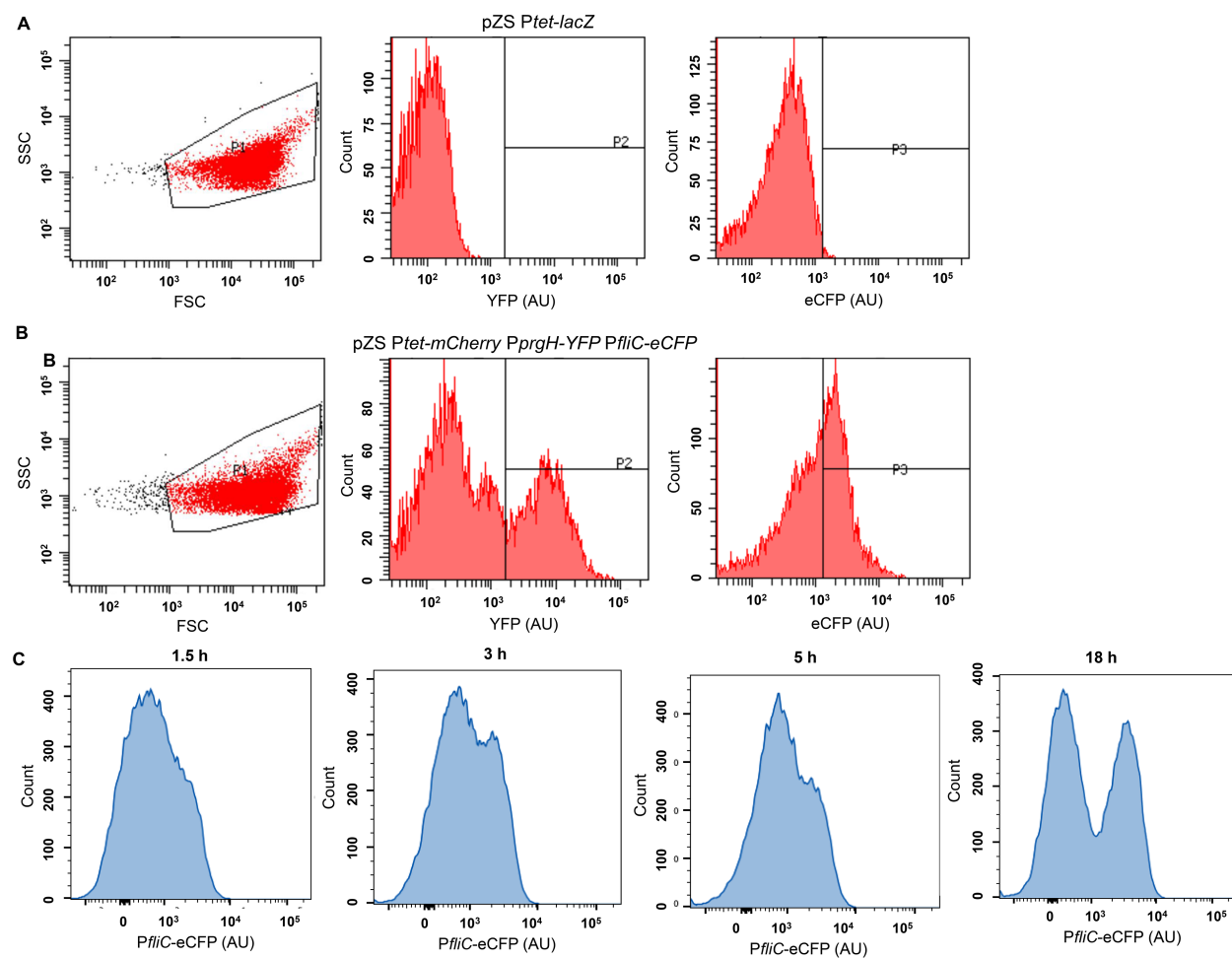

Figure S1. Lyu et al.

Supplement: FIG S1 [file mbio.02374-21-sf001.pdf]

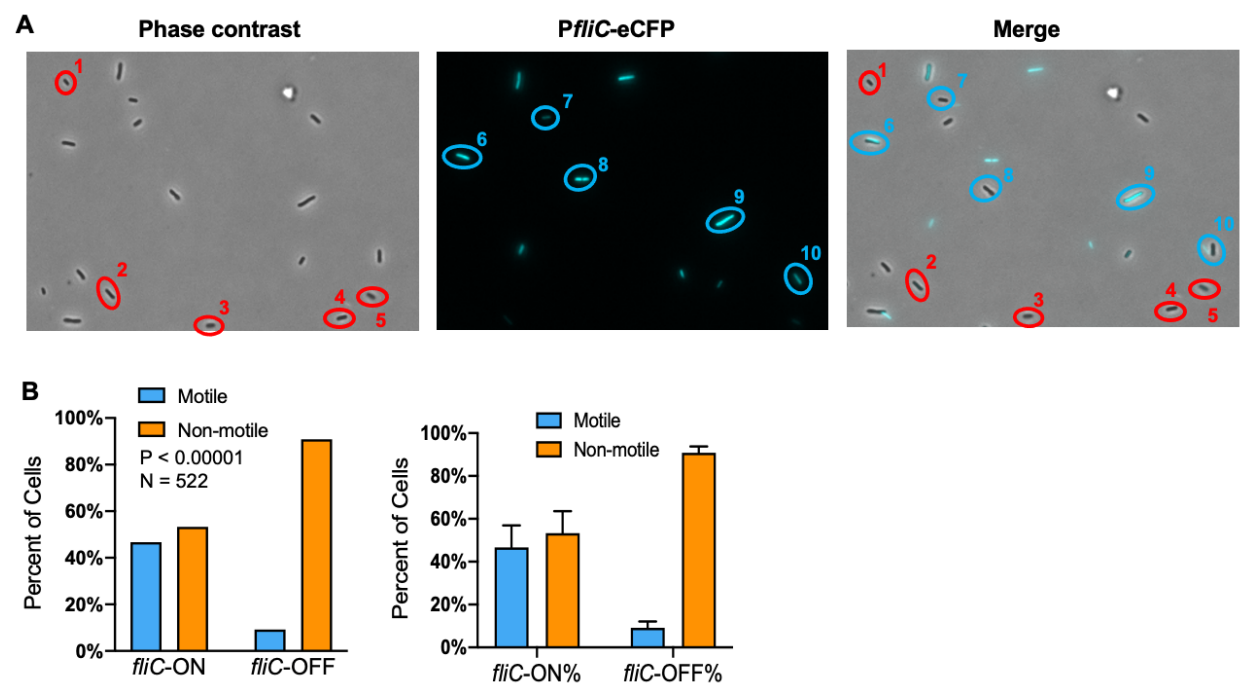

Figure S2. Lyu et al.

Supplement: FIG S2 [file mbio.02374-21-sf002.pdf]

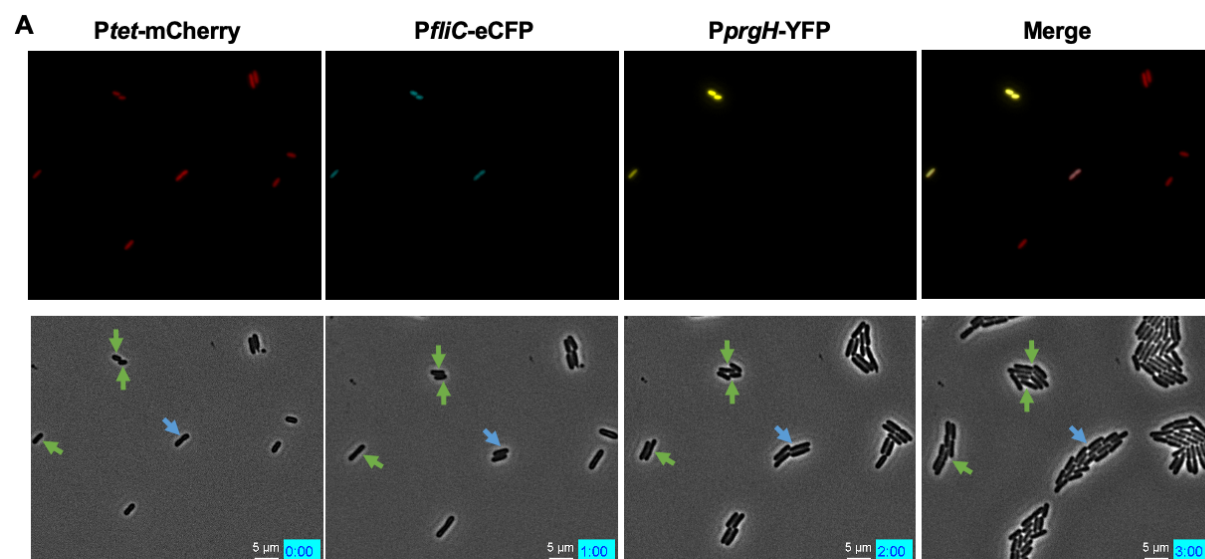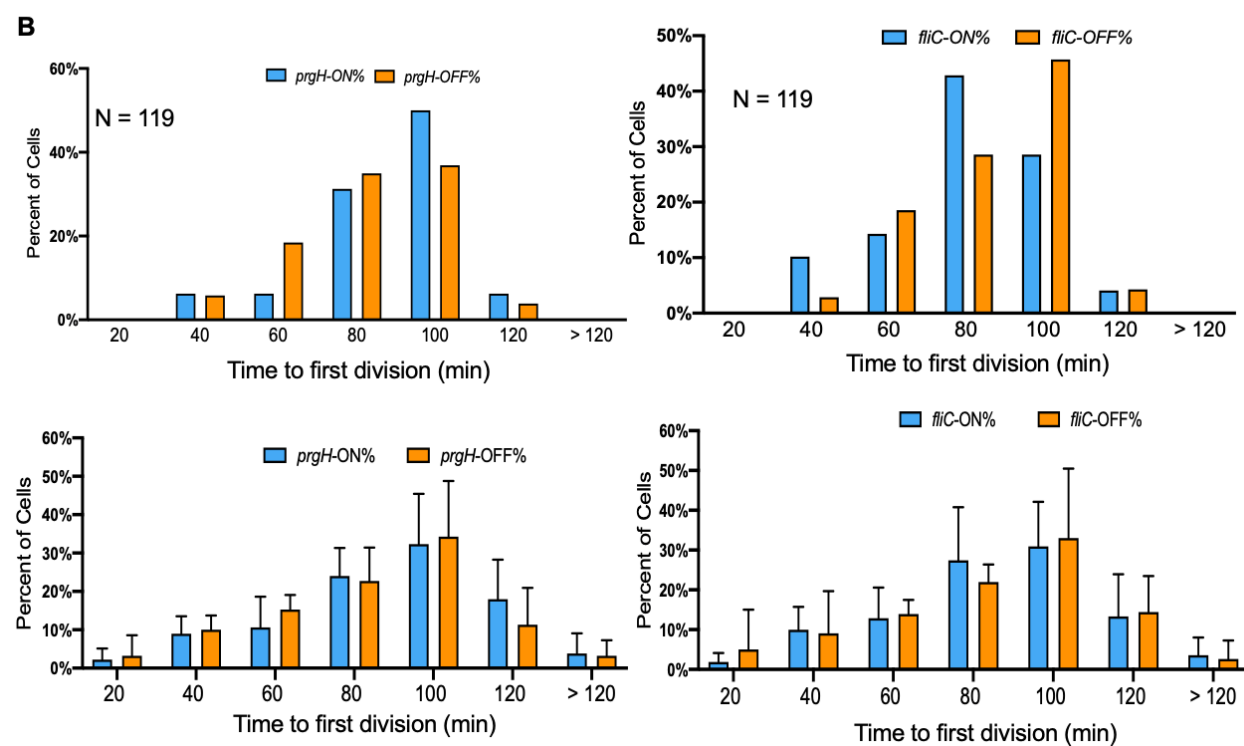

Figure S3. Lyu et al.

Supplement: FIG S3 [file mbio.02374-21-sf003.pdf]

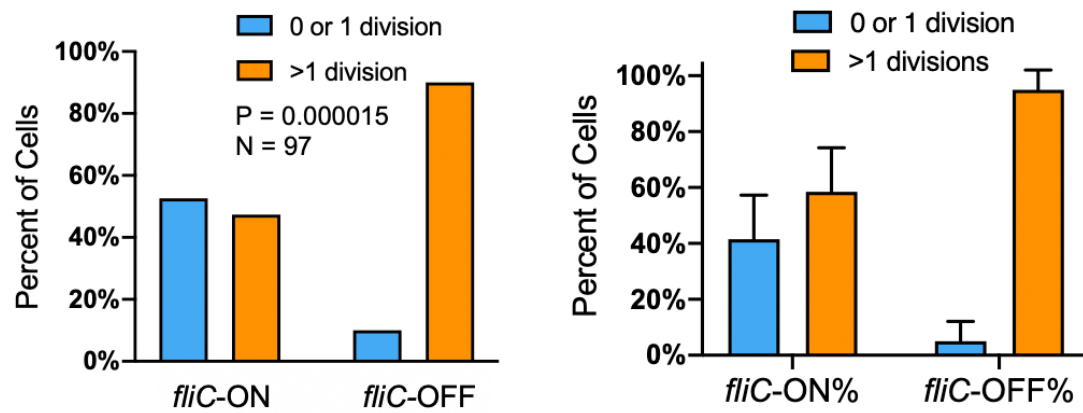

Figure S4. Lyu et al.

Supplement: FIG S4 [file mbio.02374-21-sf004.pdf]

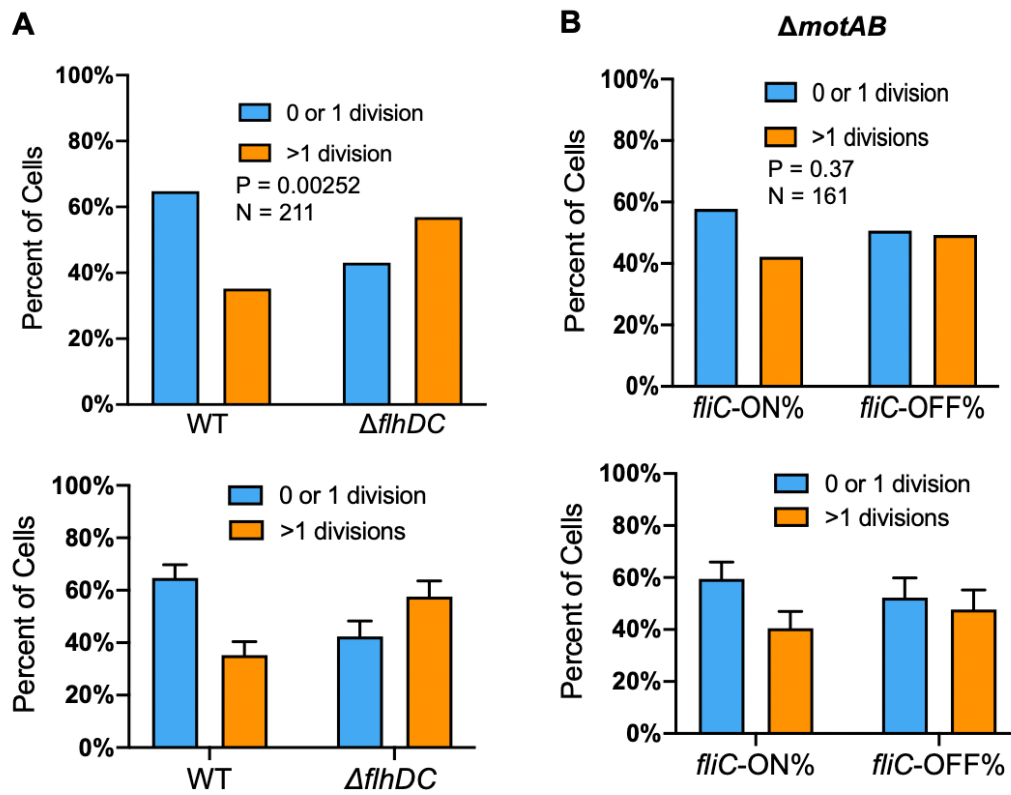

Figure S5. Lyu et al.

Supplement: FIG S5 [file mbio.02374-21-sf005.pdf]

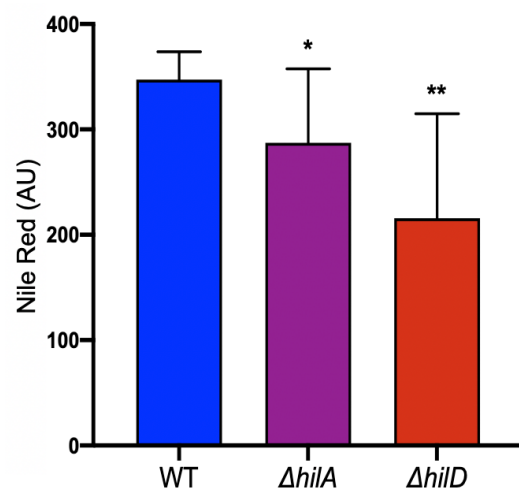

Figure S6. Lyu et al.

Supplement: FIG S6 [file mbio.02374-21-sf006.pdf]

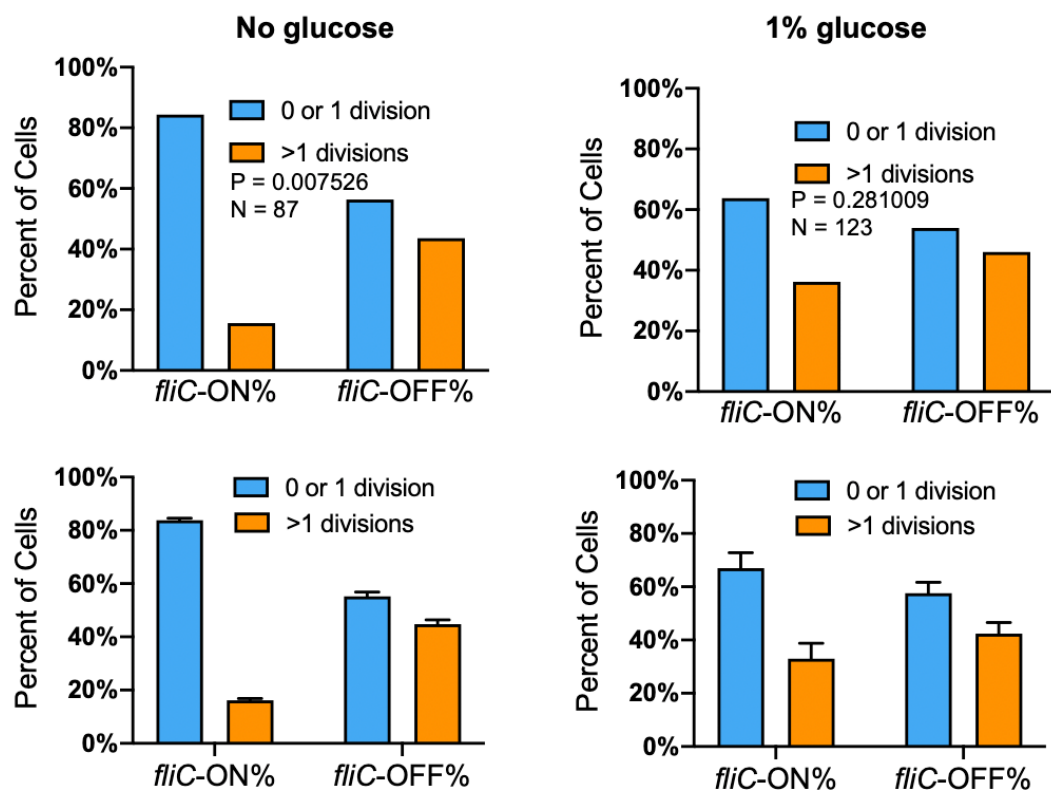

Figure S7. Lyu et al.

Supplement: FIG S7 [file mbio.02374-21-sf007.pdf]
